# Personal Agency in Borderline Personality Disorder: The Impact of Adult Attachment Style

**DOI:** 10.3389/fpsyg.2021.669512

**Published:** 2021-06-24

**Authors:** Talia Hashworth, Samantha Reis, Brin F. S. Grenyer

**Affiliations:** School of Psychology and Illawarra Health and Medical Research Institute, University of Wollongong, Wollongong, NSW, Australia

**Keywords:** personal agency, locus of control, borderline personality disorder, attachment, psychopathology, personality traits, mental health

## Abstract

**Background:**

Personal agency- the degree to which one believes they have control over their life- is thought to influence how people understand their interpersonal relationships. Links between adult attachment and personal agency are theoretically relevant to the experience of borderline personality disorder (BPD) but this has yet to be empirically examined. The present research examines the impact of personal agency and adult attachment styles for individuals meeting criteria for BPD.

**Methods:**

Participants consented to an online community study examining measures of locus of control (as an index of personal agency), BPD, and adult attachment. Participants meeting criteria for BPD (*N* = 96; mean age = 30.63; 70.5% female) were compared to age-matched healthy controls (*N* = 96; mean age = *M* = 31.99; 89.0% female).

**Results:**

Individuals who met criteria for BPD displayed lower personal agency and higher fearful and preoccupied attachment styles in their close relationships, compared to Controls. Controls reported greater personal agency and were more securely attached in their relationships. Using multiple mediation modeling, the indirect effect of personal agency on BPD was significant for preoccupied, fearful, and secure attachment, but was non-significant for dismissive attachment. Lower personal agency was associated with insecure adult attachment styles.

**Conclusions:**

Findings highlight the previously unexplored relationship between BPD and personal agency and indicate that adult attachment style plays a significant role. Low personal agency may increase challenges for individuals with symptoms of BPD by exacerbating relationship difficulties. People in treatment for BPD may benefit from focusing on both relationship insecurity and its impact on their perceived personal control.

## Introduction

Borderline Personality Disorder (BPD) is a mental disorder characterized by a pattern of impulsivity, as well as instability in interpersonal relationships, self-image, and affect (American Psychiatric Association (APA), 2013; Hashmani, 2017). People diagnosed with BPD may report frantic efforts to avoid real or imagined abandonment, unstable and intense interpersonal relationships, identity disturbances, impulsivity, suicidality or self-mutilating behaviors, affective instability, chronic feelings of emptiness, intense and inappropriate anger, and stress-related paranoia or dissociative symptoms (American Psychiatric Association (APA), 2013). Prevalence rates in the general population are estimated to be between 1 and 5.9% (Lieb et al., 2004; American Psychiatric Association (APA), 2013) but can affect up to 20% of all psychiatric inpatients (Witt et al., 2017). Compounding the clinical picture, research suggests that individuals with BPD tend to report lower personal agency, meaning they believe that they have little control over themselves and their environment. This may lead to further symptoms of psychopathology, difficulty managing life stressors, poor emotion regulation, insecure attachment styles, and challenges engaging in and sustaining treatment (Gold and Kyratsous, 2017; Flaherty, 2018; Hope et al., 2018; Moreira et al., 2020).

The concept of personal agency has become more relevant for researchers and clinicians alike (Groth et al., 2019), particularly since personal agency has been shown to impact treatment outcome (Lefcourt and Davidson-Katz, 1991; Bateman et al., 2015). Within research, personal agency may be operationalized as locus of control (LOC) (Tyler et al., 2020), a related construct that underlies how individuals explain events, situations and interactions in their lives. From this perspective, high personal agency maps on to an internal LOC—the idea that outcomes are dependent upon the effort individuals expend in their pursuit. Individuals reporting internal LOC believe that outcomes are generally contingent upon the work put into them and are more likely to apply their efforts in important tasks (Lefcourt and Davidson-Katz, 1991). Conversely, low personal agency maps conceptually onto the other extreme, external LOC, where outcomes are thought to be attributed to luck, fate, or chance, lacking a level of personal control and autonomy (Lefcourt and Davidson-Katz, 1991). Individuals reporting greater personal agency are suggested to be more active, alert, or directive in attempting to control their environment than those exhibiting less personal agency (Phares, 1976). On the other hand, individuals reporting low personal agency may fail to perceive the connections between efforts and outcomes and may perceive success as a function of luck or of being related to the “right people” than it is a result of effort or ability (Lefcourt and Davidson-Katz, 1991).

Previous studies have examined increases and decreases in locus of control through measuring personal agency, while other studies have used the term locus of control and personal agency as synonymous or as co-dependent concepts (Lamanna, 2000; Wu et al., 2015). One study noted increases in personal agency were triggered by locus of control (De Hoogh and Den Hartog, 2009), while others deemed terms such as “personal control” and locus of control synonymous (Hanes and Wild, 1977; Coleman et al., 1999). Given these relationships within present research, LOC will be utilized as an operationalization of personal agency.

Findings from the relationship between personal agency and BPD symptoms indicate that greater BPD symptom severity tends to be related to low personal agency (Watson, 1998; Hope et al., 2018). Whilst recent research examining BPD and personal agency is scarce, one older study examined the relationship between personal agency and traits of personality disorders (Watson, 1998). Individuals with BPD symptoms were distinguished from individuals with other personality disorder symptoms by lower agency and greater beliefs in the influence of external factors (Watson, 1998). In addition, maladaptive emotional regulation partially explained the link between low personal agency and BPD features (Hope et al., 2018).

Other research has compared levels of personal agency for individuals with BPD and those with depression. Findings indicated that whilst both groups reported low personal agency, there was no significant difference between the two (Pinto et al., 1996). However, within this study the severity of depression was not accounted for, individuals with comorbid BPD and depression were not excluded, and appropriate controls were not used. Conclusions specific to diagnosis should therefore be considered with caution (Pinto et al., 1996; Hope et al., 2018).

Insecure attachment styles (Bowlby, 1973; Bartholomew and Horowitz, 1991) are also common for individuals with BPD, with previous literature suggesting that the diagnosis of BPD stems from early attachment difficulties (Fonagy et al., 2000; Beeney et al., 2017; Godbout et al., 2019). According to the Psychodynamic Diagnostic Manual (PDM-2) (Lingiardi and McWilliams, 2017), various dimensions of mental functioning emerge from early relationships with primary caregivers and are associated with one’s subjective sense of internal control (i.e., personal agency), along with capacities for emotional regulation, mentalization, and one’s relationship with self and other in adulthood. When assessing mental functioning, the PDM-2 examines one’s capacity for self-esteem regulation and the quality of their internal experience by examining two core areas: one’s level of confidence in relationships with the self and others (i.e., attachment), and one’s degree of internal control, self-efficacy, and agency (i.e., personal agency) (Lingiardi and McWilliams, 2017; Lingiardi et al., 2018). From this lens, both quality of attachment and personal agency are viewed as core aspects of mental functioning within the PDM-2 (Lingiardi and McWilliams, 2017; Lingiardi et al., 2018).

Adults are thought to present with a secure attachment style or one of three insecure attachment styles: preoccupied, fearful, and dismissive (Bartholomew and Horowitz, 1991). Secure attachment indicates a sense of worthiness plus an expectation that others in close relations are generally accepting and responsive (Bartholomew and Horowitz, 1991). Preoccupied attachment involves a sense of unworthiness of self, combined with a positive view of others, such that individuals strive to achieve self-acceptance through being accepted and valued by others (Bartholomew and Horowitz, 1991). Fearful attachment similarly indicate a sense of unworthiness of self; however, such individuals also believe that others are untrustworthy and rejecting, and may avoid relationships out of fear of rejection (Bartholomew and Horowitz, 1991). Finally, the dismissive attachment style is comprised of a sense of worthiness of the self, combined with a negative view of others, and presents as avoidance of close relationships, and maintenance of independence and invulnerability as a way to protect themselves from disappointing relationship experiences (Bartholomew and Horowitz, 1991).

Research has indicated that individuals with BPD are predominately characterized by fearful and/or preoccupied attachment styles; however, rates differ among studies (Fonagy et al., 2003; Dozier et al., 2008; Scott et al., 2013; Buchheim et al., 2017). A review of 13 empirical studies showed an association between BPD and insecure attachment in adults (Agrawal et al., 2004) and these results have been replicated with participants presenting with other personality disorders (Barone et al., 2011; Scott et al., 2013). Some studies (Scott et al., 2013) have indicated that people with BPD tend to be characterized by a preoccupied attachment style, others (Barone, 2003) have implicated the fearful attachment style is prominent in the experience of BPD, whereas many have suggested a pattern of simultaneous preoccupation and fearfulness (Main, 2000; Fonagy and Bateman, 2008; Choi-Kain et al., 2009; Smith and South, 2020). Choi-Kain et al. (2009) examined self-report ratings on attachment style using the Relationship Questionnaire (RQ) (Bartholomew and Horowitz, 1991) in BPD, depression, and non-borderline comparison groups. Results indicated that the RQ self-reports were effective in replicating previous studies as the BPD participants reported higher scores on both preoccupied and fearful attachment styles than both the depression and non-borderline comparison groups (Choi-Kain et al., 2009). Studies have also demonstrated that security in attachment is associated with reduced BPD symptomology (Deborde et al., 2012; Smith and South, 2020).

In relation to personal agency, research has demonstrated an association between fearful attachment and poorer personal agency (Dilmac et al., 2009; Ravitz et al., 2010) and secure attachment associated with greater personal agency (Mickelson et al., 1997; Hexel, 2003). Despite research linking both personal agency and adult attachment to BPD, no known studies have examined these factors concurrently to explore their influence. The present study aims to provide a greater understanding of the relationship between personal agency and BPD symptoms through the lens of adult attachment. We predicted that individuals meeting criteria for BPD will exhibit lower personal agency and a propensity toward preoccupied and/or fearful attachment than Controls. Extending on this, it was predicted that the link between low personal agency and BPD would be explained (mediated) by adult attachment styles, specifically that low personal agency may predict BPD symptoms due to the influence of insecure adult attachment.

## Materials and Methods

### Participants

Ethics approval was received by the Human Research Ethics Committee on 03/12/2019, Ethics Number: 2019/374. The inclusion criteria for the study were as follows: 1. Adult (18+ years). 2. Provide informed consent to participate. 3. Provide complete responses (full data set). 4. Provide valid responses (data was excluded for participants who showed random completion patterns or failed to successfully complete two or more directed-response items to identify inattentive responding—e.g., “Please record this statement as ‘slightly agree.”’ 5. Meet inclusion/exclusion criteria for two groups. For the BPD sample, participants must meet both MSI-BPD criteria (score of 7 or higher) and BPDCL criteria (score of 100 or higher). For the Control sample participants must have scores on MSI-BPD of 6 or lower, and BPDCL scores below 62.

Calls to participate were made to mental health online forums. Of 669 people who participated in the survey, 337 participants were eligible for inclusion. From this, 192 participants met criteria for inclusion into either the BPD or Control sample, while 145 (43.0%) of the participants did not meet the criteria for BPD or Control i.e., had some symptoms of BPD or were not healthy controls. Table 1 shows the demographics of the included sample.

**TABLE 1:** Demographics and percentages for BPD sample (*N* = 96) and Control Sample (*N* = 96).

		BPD		Control		Total	
Demographic	Item	N	Percentage	N	Percentage	N	Percentage
Age		87	*M* = 30.63, *SD* = 10.04	77	*M* = 31.99, *SD* = 11.57	164	*M* = 31.27, *SD* = 10.76
Gender	Female	67	70.5%	81	89.0%	148	79.6%
	Male	23	24.2%	9	9.9%	32	17.2%
	Other Gender	5	5.3%	1	1.1%	6	3.2%
Residential country	Australia	22	23.2%	45	49.5%	67	36.0%
	Canada	5	5.3%	11	12.1%	16	8.6%
	Other Countries	16	16.8%	10	11.0%	26	14.0%
	United Kingdom	21	22.1%	14	15.4%	35	18.8%
	United States	31	32.6%	11	12.1%	42	22.6%
In a relationship	No (Single)	54	56.3%	22	24.2%	76	40.6%
	Yes	42	43.8%	69	75.8%	111	59.4%
Relationship status	*De facto*	14	14.6%	17	18.7%	31	16.6%
	Divorced	7	7.3%	2	2.2%	9	4.8%
	In a relationship but not living together	12	12.5%	22	24.2%	34	18.2%
	Married	11	11.5%	31	34.1%	42	22.5%
	Separated	5	5.2%	1	1.1%	6	3.2
	Single/None of the Above	44	45.8%	18	19.8%	62	33.2%
	Widowed	3	3.1%	0	0%	3	1.6%
Highest level of education	High school certificate or diploma	34	35.4%	6	6.6%	40	21.4%
	College/TAFE	24	25.0%	5	5.5%	29	15.5%
	University Bachelor’s Degree	19	19.8%	40	44.0%	59	31.6%
	Postgraduate Degree	13	13.5%	37	40.7%	50	26.7%
	Other	6	6.3%	3	3.3%	9	4.8%
Who do you live with?	Alone	15	15.6%	8	8.8%	23	12.3%
	Alone with child(ren)	3	3.1%	1	1.1%	4	2.1%
	Friends or housemates	17	17.7%	13	14.3%	30	16.0%
	Other	3	3.1%	2	2.2%	5	2.7%
	Other relatives	3	3.1%	2	2.2%	5	2.7%
	Parents	25	26.0%	18	19.8%	43	23.0%
	Spouse/partner	20	20.8%	30	33.0%	50	26.7%
	Spouse/partner and child(ren)	10	10.4%	17	18.7%	27	14.4%
Do you have children?	No	73	76.0%	66	72.5%	139	74.3%
	Yes	23	24.0%	25	27.5%	48	27.5%
If yes, do your children live with you?	No	10	43.5%	5	20.0%	15	31.1%
	Yes	13	56.5%	20	80.0%	33	68.8%

Eighty-seven of the ninety-six participants in the BPD sample self-reported their age (*M* = 30.63, *SD* = 10.04; range 18–60) and 95 self-reported their gender (70.5% female). The residential country of participants in the BPD sample was predominately (77.9%) from United States, Australia, and the United Kingdom. Other countries included Canada, France, South Africa, China, India, Indonesia, Malaysia, and Germany. Seventy-seven of the ninety-six participants in the Control sample self-reported their age (*M* = 31.99, *SD* = 11.57; range 19–63) and 91 self-reported their gender (89.0% female). Similarly, the residential country of participants in the Control sample was predominately (77%) from United States, Australia, and the United Kingdom.

### Measures

#### BPD Symptoms

Presence and severity of BPD symptoms were assessed using the McLean Screening Instrument for Borderline Personality Disorder (MSI-BPD) (Zanarini et al., 2003) and the BPD Checklist (BPDCL) (Bloo et al., 2017). A cut-off score of 7 or higher on the MSI-BPD indicates a likely diagnosis of BPD and suggests high sensitivity (0.81) and specificity (0.85). The internal consistency of the MSI-BPD was assessed using Cronbach’s α and indicated *a* = 0.74 (*SE* = 0.03, 95% *CI* = 0.68–0.81) (Feldt, 1965).

The Borderline Personality Disorder Checklist (BPDCL) is a 47-item self-report questionnaire based on the DSM-IV and DSM-5 criteria. The functional cut-off for non-clinical controls is reported at 62.47 (Bloo et al., 2017) and scores of 100 are consistent with a diagnosis of BPD (Bloo et al., 2017). In the current study, reliability estimates (Cronbach’s α) for the (BPDCL) were α = 0.92, indicating excellent reliability.

#### Personal Agency

To measure levels of personal agency in relation to beliefs about mental health, the Mental Health Locus of Control Scale (MH-LOC) was used (Hill and Bale, 1980). Scores (22–132) were reported on a bipolar continuum to fall between two extremes: internal locus of control extreme (i.e., high levels of personal agency) and external locus of control extreme (i.e., low levels of personal agency). Low scores reflect a more internal locus of control, while high scores reflect a more external locus of control. In the original study (Hill and Bale, 1980), the mean scores were 74.14 (*SD* = 11.19), α = 0.84. In the current study, reliability estimates (Cronbach’s α) for the MH LOC were α = 0.76, indicating acceptable reliability.

#### Adult Attachment

The Relationship Questionnaire (RQ) assessed participants’ self-reported attachment style by asking participants to read four statements and rate how applicable each item is to them (Bartholomew and Horowitz, 1991). Studies have indicated mixed findings toward the stability of the RQ attachment styles over time (Scharfe and Bartholomew, 1994; Ravitz et al., 2010). Several studies utilize the RQ as a self-report measure to assess attachment in BPD (Choi-Kain; Levy et al., 2005), and its validity is well-documented (Ravitz et al., 2010).

## Results

First, demographic information was compared between groups to assess for significant differences. Table 1 listed descriptive statistics for all categorical variables. Independent samples *t*-tests indicated no significant differences in age between groups (*t* = 0.80, *p* = 0.23). Table 1 illustrated mean scores and standard deviations among the variables of interest between the BPD sample and the Control sample. Results indicated that on average, individuals in the BPD sample displayed significantly lower personal agency (i.e., a more external LOC), greater severity of BPD symptoms, lower attachment security and higher preoccupied and fearful attachment than controls.

Chi square tests of association illustrated that there was a significant association between group and: gender (*X*^2^ = 10.04, *df* = 2, Φ = 0.23, *p* < 0.01), relationship (single vs. in a relationship) (*X*^2^ = 19.92, *df* = 6, Φ = 0.33, *p* < 0.01), highest level of education (*X*^2^ = 51.95, *df* = 4, Φ = 0.53, *p* < 0.01), and residential country (*X*^2^ = 22.38, *df* = 4, Φ = 0.35, *p* < 0.01). Phi effect sizes suggest a small effect of gender, medium effect of residential country, and medium-large effects for relationship status and level of education.

In Table 2, independent samples *t*-tests illustrated that there were significant differences between the two groups on secure, fearful, and preoccupied attachment styles (*p* < 0.01). There were no significant differences between groups on dismissive attachment. Cohen’s *d* and Pearson’s *r* values indicated large differences between groups for all variables except for dismissive attachment. Results indicated that fearful attachment had the largest mean difference in scores between groups (−4.48), followed by secure (4.00), preoccupied (−4.03), and dismissive attachment (0.42).

**TABLE 2:** Means, standard deviations, and *T*-test for variables in BPD (*N* = 96) and Control Sample (*N* = 96).

	BPD		Controls						
Measure	Mean	SD	Mean	SD	t	df	*p*-value	Cohen’s *d*	Effect-size *r*
MH LOC	79.03**	11.39	71.96	12.35	–4.13	190	0.00	0.60	0.29
BPD Checklist	136.51**	23.36	55.20	4.43	–33.51	190	0.00	4.86	0.92
Secure attachment	3.34**	2.37	7.34	2.65	11.03	190	0.00	1.60	0.62
Fearful attachment	9.10**	2.18	4.63	2.81	–12.34	190	0.00	1.79	0.67
Preoccupied attachment	7.40**	3.50	3.36	2.37	–9.36	190	0.00	1.36	0.56
Dismissive attachment	5.23	3.08	5.65	3.22	0.92	190	0.36	0.13	0.07

**p < 0.01.

Pearson correlations were utilized to examine the relationship between personal agency and BPD symptoms (BPDCL). Results within the full sample (*N* = 192), regardless of group, indicated that personal agency has a positive and significant relationship with BPD symptoms (*r* = 0.34, *p* < 0.01).

To control for education and relationship status on the relationship between personal agency and BPD, an ANCOVA was used. Results indicate that when these demographic variables were controlled for, the relationship between personal agency and BPD remained significant, suggesting that differences in demographics did not significantly affect the results.

Next, correlational analyses were used to understand relationships between personal agency and attachment. Results from the full sample illustrate significant associations between personal agency and all attachment styles [Secure (*r* = −0.24), Fearful (*r* = 0.34), Preoccupied (*r* = 0.19), and Dismissive (*r* = 0.19), *p* < 0.01]. Low personal agency was significantly and negatively related to secure attachment, and positively and significantly related to fearful, preoccupied, and dismissive attachment.

The secure (*r* = −0.62, *p* < 0.01), preoccupied (*r* = −0.65, *p* < 0.01), and fearful (*r* = −0.57, *p* < 0.01) attachment styles were all significantly correlated with BPD symptoms as measured by the BPDCL. Dismissive attachment (*r* = −0.09) was not significantly correlated with the BPDCL.

**FIGURE 1:**
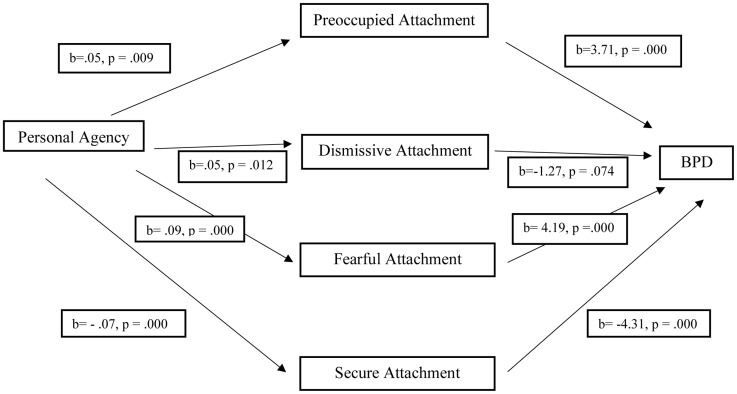
Multiple mediation analysis of personal agency to BPD through four attachment styles.

A multiple mediation analysis using PROCESS v3.4.1 with 5,000 samples of bootstrapping was conducted to examine the relationship between personal agency and BPD and whether these were mediated by attachment styles (preoccupied, dismissive, fearful, and secure), illustrated by Figure 1. The relationship between personal agency and preoccupied (*b* = 0.05, *SE* = 0.021, *p* = 0.009), fearful (*b* = 0.09, *SE* = 0.019, *p* = 0.000), and dismissive attachment (*b* = 0.05, *SE* = 0.018, *p* = 0.012) was positive and significant, and secure attachment was negative and significant (*b* = −0.07, *SE* = 0.018, *p* = 0.000). The relationship between BPD and preoccupied (*b* = 3.71, *SE* = 0.672, *p* = 0.000), fearful (*b* = 4.19, *SE* = 0.865, *p* = 0.000), and secure (*b* = −4.31, *SE* = 0.817, *p* = 0.000) attachment was significant, while dismissive attachment was not significantly associated with BPD (*b* = −1.27, *SE* = 0.705, *p* = 0.074). Secure attachment accounted for approximately 69% of the variance in personal agency and BPD [*F*_(__1, 190)_ = 14.09, *p* = 0.000, *R*^2^ = 0.069], followed by preoccupied with 35% of the variance [*F*_(__1, 190)_ = 6.89, *p* = 0.009, *R*^2^ = 0.035], dismissive with 32% of the variance [*F*_(__1, 190)_ = 6.50, *p* = 0.012, *R*^2^ = 0.033], and fearful with 12% of the variance [*F*_(__1, 190)_ = 28.82, *p* = 0.000, *R*^2^ = 0.116]. The direct effect of personal agency on BPD was not significant, *b* = 0.20, *p* = 0.25, 95%CI [−0.15, 0.56]. The indirect effect of personal agency on BPD was significant for preoccupied attachment, fearful attachment, and secure attachment, and was not significant for dismissive attachment.

Direct effect of personal agency on BPD: *b* = 0.20, *p* = 0.25, 95%CI [−0.15, 0.56].

Indirect effects of personal agency on BPD through preoccupied attachment: *b* = 0.20, 95%CI [0.04, 0.39].

Indirect effects of personal agency on BPD through dismissive attachment: *b* = −0.06, 95%CI [−0.17, 0.01].

Indirect effects of personal agency on BPD through fearful attachment: *b* = −0.39, 95%CI [0.20, 0.63].

Indirect effects of personal agency on BPD through secure attachment: *b* = 0.29, 95%CI [0.12, 0.48].

## Discussion

This study was the first to simultaneously examine personal agency (operationalized as locus of control), BPD symptomology and adult attachment styles. Findings indicated that low personal agency was associated with greater BPD symptomology, negatively related to secure attachment and positively related to fearful, preoccupied, and dismissive attachment. Conversely, high personal agency was related to low BPD symptomology and attachment security. Participants with BPD were significantly more likely to report low personal agency and fearful and preoccupied attachment styles than Controls. These results indicate that individuals with fewer BPD symptoms and those with more secure attachments tend to show greater personal agency. A possible explanation is that individuals with fewer BPD symptoms and greater attachment security play a more active role in their treatment and are more likely to experience positive treatment outcomes. According to the PDM-2, quality of attachments and personal agency are viewed as core aspects of mental functioning for individuals with BPD (Lingiardi and McWilliams, 2017; Lingiardi et al., 2018).

Results of multiple mediation modeling demonstrated that attachment styles explained the link between personal agency and BPD symptomology. The preoccupied and fearful attachment styles may therefore be the mechanism by which low personal agency may lead to more severe BPD symptomology. Conversely, the secure attachment style may provide a buffer against experiencing BPD symptoms when one perceives themselves as having little control over their life. Since this is the first research to test this association, results are tentative and should aim to be replicated using a clinical sample of individuals diagnosed with BPD.

Consistent with previous research (Hexel, 2003; Dilmac et al., 2009; Di Pentima et al., 2019), the pattern of relationships between personal agency and secure attachment differs from the other attachment styles, as increased attachment security appears to contribute to high personal agency. A potential explanation would be that attachment styles featuring a negative model of self (fearful and preoccupied) may lead those who perceive a lack of control over themselves and their environment to experience high levels of dysregulation and maladaptive coping strategies, often seen in BPD. Once all potential attachment mediators were considered simultaneously, dismissive attachment was not associated with BPD but was positively associated with personal agency. These results propose that attachment can explain the link between low personal agency and high BPD symptomology.

The present research has implications for therapy of individuals experiencing BPD. These findings highlight the importance of understanding BPD symptoms, by viewing them through a lens of low to high personal agency. The results of this study along with theoretical underpinnings from the PDM-2 suggest that personal agency is one of the key factors in influencing BPD symptomology. It may be therefore be that targeting levels of personal agency in patients (through increasing autonomy), and simultaneously targeting attachment problems may be beneficial. It is known that individuals with BPD tend to have low personal agency, but this study suggests that adult attachment styles may explain this relationship. Increasing personal agency, autonomy, and perceived control, and providing a foundation for development of more secure attachments during therapy may improve outcomes for patients with BPD.

It is recommended that clinicians actively work toward increasing levels of personal agency by promoting mentalization, managing cognitive distortions (including self-blame and blaming others), increasing self-compassion, challenging unhelpful thinking styles that limit personal agency, and encouraging clients to play an active role in therapy. This may include collaboratively deciding treatment approaches and allowing clients to initiate booking follow-up sessions. By allowing clients to take a larger role in their treatment, the client may generalize this experience to other areas of their life, leading to a broader improvement in their sense of agency. Considering the complexity in working with BPD, it is hoped that clinicians implement strategies to directly target personal agency and assist individuals with BPD to improve their sense of control over their lives.

A strong therapeutic alliance, confidence to make and follow through with decisions, an active role in treatment, and validation of therapeutic achievements, both large and small, may improve the efficacy of therapy for BPD clients. Findings from this study may benefit clinicians and suggest that conscious efforts to increase clients’ personal agency through treatment may lead to positive outcomes. Individuals with BPD may struggle with their self-critical voice and experience self-doubt; however, effective therapeutic outcomes may be achieved with a greater focus on teaching skills to manage high levels of distress independently (without depending on the therapist), increasing one’s self-worth, trust in self, and motivation to take steps to manage their mental health, thereby improving personal agency. From this, these findings have important implications for understanding how the therapeutic alliance may play a role in encouraging individuals with BPD to take more agency. Our findings suggest that addressing levels of personal agency and relationship insecurity may assist in managing some of the core underlying difficulties with people with BPD—dysfunction and dysregulation of the relationship between self and others. Such concerns are the cornerstone to understanding and treating BPD and interventions targeting personal agency may assist in alleviating such challenges.

### Limitations

There were some limitations in the present study. First, due to the nature of an online and anonymous study, it was not possible to follow up with participants after completion. Thus, we did not conduct a clinical diagnostic interview to confirm clinical status. To manage this limitation, strict inclusion criteria were required, increasing the likelihood that participants in the BPD sample present with diagnostic criteria for BPD and ensuring participants met cut offs from both instruments to be included. However, despite efforts to ensure strict inclusion criteria, we were unable to guarantee the exclusion of participants with comorbid disorders. It is possible that participants in the BPD group may present with symptoms of BPD alongside other diagnoses, and this may impact the results.

Second, though we do not anticipate that the differences in demographics were clinically significant, it is possible that such differences may have impacted the results. Analyses indicated a small effect of gender, medium effect of residential country, and medium-large effects for relationship status and level of education, suggesting that differences in gender and residential country likely did not contribute to the results. To examine the effect of level of education and relationship status, both variables were controlled for in the relationship between personal agency and BPD. Results indicated that neither of these variables influenced the results. In addition, the groups were identical in numbers and did not present with any significant differences in regard to their age. This study utilized participants from various countries, meaning cultural differences in BPD may be present but were not examined or assessed for in the present study. To assess for cultural differences in BPD, future research should investigate cross-cultural differences among both Eastern and Western countries.

Third, the study used a cross-sectional design and therefore only assessed participants at one time point. Participants were divided into groups based on whether or not they met threshold criteria for BPD at the time of the survey. It is unclear whether participants exhibited changes in their symptoms following the survey. In addition, the study utilized self-report measures, as opposed to clinical diagnostic interviews. As with all studies that use self-report measures, caution should be taken when interpreting the results. Though we are aware of criticism around the use of the self-report (as opposed to interview-based) measures of attachment, the RQ was the most appropriate tool to assess for attachment styles in our sample, due to its conceptual link to Bartholomew and Horowitz’s (1991) adult attachment theory and its brevity. We also chose to use this measure due to its effectiveness for assessing adult attachment style in previous research (Levy et al., 2005; Choi-Kain et al., 2009; Badoud et al., 2018). For example, Choi-Kain et al. (2009) used the RQ and self-report questionnaires among a sample of BPD, with results replicating other studies that used clinical interviews to assess attachment in BPD (Barone et al., 2011). Future research may benefit from examining personal agency in a clinical population with BPD patients, utilizing a longitudinal design with follow up points and a combination of both self-report and interview-based measures.

Fourth, the MH LOC scale was a core focus of the present study; however, this scale has been minimally used in previous literature. Given this, there is a lack of knowledge and psychometric properties around its use, including reliability estimates. The most frequently used instrument to measure personal agency is Rotter’s (1966) Internal-External (I-E) Locus of Control Scale; however, there are limitations of using this measure, including that it is overly global (rather than specific) and limited in response choices, and is thus less suitable for clinical populations with high mental health presentations (Lefcourt and Davidson-Katz, 1991). Furthermore, Rotter (1975), Phares (1976), and Lefcourt (1976) recommended the development of area-specific instruments in order to assist with practical application. For this reason, the MH LOC was developed to specifically target control as it relates to mental health needs, improving relevance to clinical research.

Future research should build upon the present study by providing further validation of the MH LOC scale and its psychometrics within a clinical sample. Future research is also needed to assess the impact of these factors in therapeutic settings, particularly by examining the role of the therapeutic alliance alongside personal agency and attachment styles. It may also be of interest for future research to examine the role of other related factors, like childhood trauma, and comorbid mood disorders to better understanding the role of personal agency among individuals with BPD. In accordance with this, research should examine personal agency in BPD, with and without comorbid diagnoses. These advances may improve understanding of how to work with and successfully treat individuals with BPD.

## Data Availability Statement

The data is not available as participants only gave ethical consent for this project, and not for further distribution outside the research team.

## Ethics Statement

The studies involving human participants were reviewed and approved by the Human Research Ethics Committee, Ethics Number: 2019/374. The patients/participants provided their written informed consent to participate in this study.

## Author Contributions

This research was published as part of TH’s Ph.D. Candidature. TH collected, analyzed, and reported the data. SR and BG supervised and supported TH through the entire process. All parties contributed to the present study. All authors contributed to the article and approved the submitted version.

## Conflict of Interest

The authors declare that the research was conducted in the absence of any commercial or financial relationships that could be construed as a potential conflict of interest.

## Abbreviations

BPD: Borderline Personality Disorder
BPDCL: The Borderline Personality Disorder Checklist
LOC: Locus of Control
MH LOC: The Mental Health Locus of Control Scale
RQ: The Relationship Questionnaire
MSI-BPD: The McLean Screening Instrument for Borderline Personality Disorder.
